# Quantum Central Limit Theorems, Emergence of Classicality and Time-Dependent Differential Entropy

**DOI:** 10.3390/e25040600

**Published:** 2023-04-01

**Authors:** Tien D. Kieu

**Affiliations:** Centre for Quantum Technology Theory, and Optical Sciences Centre, Swinburne University of Technology, Hawthorn, VIC 3122, Australia; tien.d.kieu@gmail.com

**Keywords:** quantum central limit theorems, emergence of classicality, time-dependent entropy

## Abstract

We derive some quantum central limit theorems for the expectation values of macroscopically coarse-grained observables, which are functions of coarse-grained Hermitian operators consisting of non-commuting variables. Thanks to the Hermiticity constraints, we obtain positive-definite distributions for the expectation values of observables. These probability distributions open some pathway for the emergence of classical behaviours in the limit of an infinitely large number of identical and non-interacting quantum constituents. This is in contradistinction to other mechanisms of classicality emergence due to environmental decoherence and consistent histories. The probability distributions thus derived also enable us to evaluate the non-trivial *time-dependence* of certain differential entropies.

## 1. Opening Remarks

The central limit theorem (CLT) [1] for sums of independent identically distributed (iid) random variables is one of the most fundamental pillars of classical probability theory. The CLT and its various generalisations [2] have found numerous applications in diverse fields including mathematics, physics, information theory, economics, finance and psychology (see [1,2] and references therein).

The CLT has also been generalised to various quantum versions [3,4,5,6,7,8,9,10,11] which have also found many applications in quantum statistical mechanics, quantum field theory, quantum information theory, graph theory, non-commutative algebras and non-commutative stochastic processes (see [11] and references therein).

In this paper, we derive a quantum version of the CLT for the expectation values of Hermitian operators only, and not those of general operators. This Hermiticity constraint for observables results in positive-definite probability distributions—in contradistinction to the Wigner function, which is a quasiprobability distribution that, despite being real-valued, is not positive-definite in general, as well as different to the Glauber–Sudarshan P representation [12,13], which is the quasiprobability distribution in which observables are expressed in normal order. Our probability distributions below, (8) and (19), are *unique* and *independent* of the operators being considered. We employ in our derivation a renormalisation blocking approach to obtain *explicit* expressions for the probability distributions. These are presented in the next three sections.

Note that, on the other hand, previous versions of quantum CLT consider general operators, including non-Hermitian ones, and thus do not explicitly express the resulting quasi-distributions but only *implicitly* through expectation values with Gaussian states. The exact forms of those quasi-distributions, as such, may also be dependent on the operators being considered.

Our explicit forms of the so-derived probability distributions afford us a pathway for the emergence of classical behaviours from the quantum mechanics of a system having non-interacting constituents when the number of constituents is taken to infinity. This is discussed in Section 5. Our pathway for an emergence of classicality is quite different from and in contrast to that afforded by decoherence and/or consistent histories [14,15,16], and other approaches such as that of GRWP [17,18], where the spontaneous collapse of wave function is approached for a many-body system of distinguishable or indistinguishable particles [19]. It should be noted that our emergence mechanism is not contradictory to, neither exclusive of, and may not replace the latter where interactions/noises are taken into account. In ours, no new and extra fluctuating stochastic field need to be introduced into the dynamics in order to cause the evolution of the state vector of a macroscopic system to “collapse”. The inherent quantum fluctuations in the wave function of individual constituents are cancelled out in the collective, block variables—even though there are no interactions among the constituents.

We then use our explicit probability distributions for the evaluation of a particular form of differential entropy for some simple quantum systems in Section 7. In the literature for both open and closed quantum systems, different information-theoretical entropy measures have been discussed [20,21]. The measure of differential entropy we employ is a special case of relative entropy, argued for based on the considerations by Jaynes [22]. In contrast to the von Neumann entropy which simply vanishes on pure states, the differential entropy quantifies the degree of probability (de)localisation and its time development [23].

The last section of the paper contains our concluding remarks.

## 2. Quantum Central Limit Theorem and Heuristic Renormalisation Blocking

Renormalisation group blocking plays a central role in understanding emerging bulk behaviours and collective phenomena. Heuristically, one could start with a path integral or partition function in some set of fundamental microscopic variables/operators. As an illustration, let us take the path integral expression for a quantum system having the action S[ξ] in the fundamental field variable ξ,

(1) $$\begin{array}{ccc} Z & = & \int \exp\{iS[\xi ]\}D\xi . \end{array}$$

In deriving coarse-graining behaviours from the system, we introduce the coarse-grained field variable Ξ as a function of the fundamental variables ξ in some chosen blocking scheme $\Xi_{j}=h\left(\xi \right)$, as in an averaging scheme, for example,

(2) $$\begin{array}{ccc} \Xi_{j} & = & \frac{1}{V_{j}}\sum_{\xi_{i}\in \mathrm{block}\;j}\xi_{i}, \end{array}$$

where $V_{j}$ is a measure of the “volume” of each block. We have to choose the blocking function *h* in such a way that the coarse-grained variables are not growing indefinitely in magnitude when we keep on successively coarse-graining the coarse-grained variables to the next level—hence the volume denominator in our example above.

The expectation value of a quantum operator 〈F(Ξ)〉 of the coarse-grained variables could then be expressed as

(3) $$\begin{array}{ccc} \langle F(\Xi (\xi ))\rangle & = & \frac{1}{Z}\int F\left(\Xi \left(\xi \right)\right)\exp\left\{iS\left[\xi \right]\right\}D\xi . \end{array}$$

To convert the last path integral in Dξ to that in DΞ, we insert the resolution of unity

(4) $$\begin{array}{ccc} 1 & = & \int \delta (\Xi -h(\xi ))D\Xi \end{array}$$

into (3), and then interchange the order of integration to obtain

(5) $$\begin{array}{ccc} \langle F(\Xi )\rangle & = & \frac{1}{Z}\int F\left(\Xi \right)\exp\left\{iS^{\prime }\left[\Xi \right]\right\}D\Xi , \end{array}$$

where

(6) $$\begin{array}{ccc} \exp\{iS^{\prime }\left[\Xi \right]\} & = & \int \delta (\Xi -h(\xi ))\exp\{iS[\xi ]\}D\xi . \end{array}$$

The successive repetition of the last expression defines a renormalisation group flow.

We will phrase the quantum central limit theorems in this paper as a restricted renormalisation blocking in the sense that we only consider the expectation values of Hermitian operators and not the full path integral/partition function for arbitrary operators.

Our restricted consideration results in positive-definite measures which can be interpreted as probability measures, from which the fixed-point distributions of the renormalisation blocking emerge.

## 3. Central Limit Theorem for Single Hermitian Variable

The centre of mass, or intensive variables in general, of a composite systems of *N* components can be expressed as

(7) $$\begin{array}{ccc} \hat{X} & = & \frac{1}{N}\left({\hat{x}}_{1}⊗{\hat{1}}_{2}\cdots ⊗{\hat{1}}_{N}+{\hat{1}}_{1}⊗{\hat{x}}_{2}\cdots ⊗{\hat{1}}_{N}+\cdots +{\hat{1}}_{1}⊗{\hat{1}}_{2}\cdots ⊗{\hat{x}}_{N}\right),\\ & \equiv & \frac{1}{N}\sum_{i=1}^{N}{\hat{x}}_{i}. \end{array}$$

We now consider a system with identical and non-interacting components (such as the case of an ideal gas)

$$|\Phi \rangle ={⊗}_{i}^{N}|\phi_{i}\rangle ,$$

where $|\phi_{i}\rangle =|\phi \rangle$, for all *i*.

With some general function *f*, we obtain the following result for N≫1

(8) $$\begin{array}{ccc} \langle \Phi \left|f\left(\frac{1}{N}\sum_{i}^{N}{\hat{x}}_{i}\right)\right|\Phi \rangle & \overset{N\to \infty }{⟶} & \frac{1}{\left(\sigma_{x}/\sqrt{N}\right)2\pi \sqrt{2\pi }}\int dXf\left(X\right)\exp\left\{-\frac{{\left(X-\langle x\rangle \right)}^{2}}{2{\left(\sigma_{x}/\sqrt{N}\right)}^{2}}\right\}, \end{array}$$

where

(9) $$\begin{array}{ccc} \langle x\rangle & \equiv & {\int x|\langle x|\phi \rangle |}^{2}=\langle \phi |\hat{x}|\phi \rangle , \end{array}$$

(10) $$\begin{array}{ccc} \sigma_{x}^{2} & \equiv & \langle x^{2}\rangle -{\langle x\rangle }^{2}. \end{array}$$

The derivation of the above is given in Appendix A.

In particular, we can derive, as a special case from above, the probability density for finding *X* around some $X_{0}$,

(11) $$\begin{array}{ccc} \langle \Phi \left|\delta \left(\hat{X}-X_{0}\hat{1}\right)\right|\Phi \rangle & ∼ & \int \delta \left(X-X_{0}\right)\exp\left\{-\frac{{\left(X-\langle x\rangle \right)}^{2}}{2{\left(\sigma_{x}/\sqrt{N}\right)}^{2}}\right\}dX,\\ & ∼ & \exp\left\{-{\left(X_{0}-\langle x\rangle \right)}^{2}/2{\left(\sigma_{x}/\sqrt{N}\right)}^{2}\right\}, \end{array}$$

which is a Gaussian distribution.

We estimate from the derivation that the size of the system should satisfy the condition $N\gg \left|\langle x^{3}\rangle /\langle x^{2}\rangle \right|$ for the approximation.

We can also easily generalise the result to the case in which the initial state is a mixed state instead of being pure.

Also note that the above result can be readily generalised to the case when

$$f\left(\frac{1}{N}\sum_{i}^{N}x_{i}\right)⟶f\left(\frac{1}{N^{m}}\sum_{i}^{N}g\left(x_{i}\right)\right),\;m\geq 1$$

where *m* is integer and g() is some arbitrary function.

In the limit of N→∞, the Gaussian distribution in (8) converges to a delta distribution,

(12) $$\begin{array}{ccc} \frac{1}{\left(\sigma_{x}/\sqrt{N}\right)2\pi \sqrt{2\pi }}\exp\left\{-\frac{{\left(X-\langle x\rangle \right)}^{2}}{2{\left(\sigma_{x}/\sqrt{N}\right)}^{2}}\right\} & \overset{N\to \infty }{⟶} & \delta (X-\langle x\rangle ). \end{array}$$

We thus have from (8), for arbitrarily finite integer *m*,

(13) $$\begin{array}{ccc} \langle \Phi \left|{\hat{X}}^{m}\right|\Phi \rangle & \overset{N\to \infty }{⟶} & \int dX\;X^{m}\delta \left(X-\langle x\rangle \right)={\langle x\rangle }^{m}. \end{array}$$

This is an indication of an emergence of classical behaviours for a macroscopically blocked variable *X*, as the right-hand side of the last expression contains ${\langle x\rangle }^{m}$ rather than $\langle x^{m}\rangle$.

In order to verify such emergence, we need to further consider quantum mechanically non-commuting variables in the next section.

## 4. A Central Limit Theorem for Non-Commuting Variables

We additionally consider the momentum operators ${\hat{p}}_{i}$, the non-commuting conjugate variables of the position operators ${\hat{x}}_{i}$, and introduce the blocked variable $\hat{P}$

(14) $$\begin{array}{ccc} \hat{P} & = & \frac{1}{N}\left({\hat{p}}_{1}⊗{\hat{1}}_{2}\cdots ⊗{\hat{1}}_{N}+{\hat{1}}_{1}⊗{\hat{p}}_{2}\cdots ⊗{\hat{1}}_{N}+\cdots +{\hat{1}}_{1}⊗{\hat{1}}_{2}\cdots ⊗{\hat{p}}_{N}\right),\\ & \equiv & \frac{1}{N}\sum_{i=1}^{N}{\hat{p}}_{i}, \end{array}$$

while $\hat{X}$ of the last section is the centre of mass, this blocked variable $\hat{P}$ corresponds to a measure of the velocity of the centre of mass. In the Heisenberg picture,

(15) $$\begin{array}{ccc} \frac{d}{dt}X & = & \frac{i}{\hbar }\left[H,X\right],\\ & = & \frac{i}{\hbar }\left[\sum_{i}^{N}\frac{p_{i}^{2}}{2m}+V_{i}\left(x_{i}\right),\frac{1}{N}\sum_{j}^{N}x_{j}\right],\\ & = & P/m. \end{array}$$

Even for the system of interacting components, we have

(16) $$\begin{array}{ccc} \left[\hat{X},\hat{P}\right] & = & \frac{1}{N^{2}}\left[\sum_{i}^{N}{\hat{x}}_{i},\sum_{j}^{N}{\hat{p}}_{j}\right],\\ & = & \frac{1}{N^{2}}\sum_{i}^{N}\left[{\hat{x}}_{i},{\hat{p}}_{i}\right]\\ & = & i\hbar /N,\\ & \overset{N\to \infty }{⟶} & 0. \end{array}$$

Now with

$$\Delta A^{2}\;\Delta B^{2}\geq {\left|\frac{1}{2}\langle \left\{\hat{A},\hat{B}\right\}\rangle -\langle \hat{A}\rangle \langle \hat{B}\rangle \right|}^{2}+{\left|\frac{1}{2i}\langle \left[\hat{A},\hat{B}\right]\rangle \right|}^{2},$$

we have, because of the approximate commutativity above,

(17) $$\begin{array}{ccc} \Delta X\;\Delta P & ∼ & O\left(1/N\right)\overset{N\to \infty }{⟶}0. \end{array}$$

We now consider a *Hermitian* combination of some finite sum of products of $\hat{X}$ and $\hat{P}$, which can be generally expressed as, by constraint of Hermiticity,

(18) $$\begin{array}{c} c_{mn}{\left(\hat{X}\right)}^{m}{\left(\hat{P}\right)}^{n}+c_{mn}^{*}{\left(\hat{P}\right)}^{n}{\left(\hat{X}\right)}^{m}, \end{array}$$

where $c_{mn}$ are c-numbers. For the expectation values of general observables, we can indeed further restrict the above to real values of $c_{mn}$.

For N≫1, we obtain the following result, of which the derivation is presented in Appendix B,

(19) $$\begin{array}{ccc} \langle \Phi \left|\sum_{mn}\left(c_{mn}X^{m}P^{n}+c_{mn}^{*}P^{n}X^{m}\right)\right|\Phi \rangle & = & \int dXdP\;\left(2\sum_{mn}ℜ\left(c_{mn}\right)X^{m}P^{n}\right)P_{re}\left(X,P\right)\\ & & +\int dXdP\;\left(\sum_{mn}ℑ\left(c_{mn}\right)X^{m}P^{n}\right)P_{im}\left(X,P\right), \end{array}$$

where the probability distribution for the real parts $ℜ(c_{mn})$ is

(20) $$\begin{array}{ccc} P_{re}\left(X,P\right) & ∼ & \exp\left\{-\frac{{\left[\left(X-\langle x\rangle \right)\cos\theta_{+}+\left(P-\langle p\rangle \right)\sin\theta_{+}\right]}^{2}}{(\sigma_{x}^{2}+\sigma_{p}^{2}+\Delta_{+})/N}\right\}\\ & & \times \exp\left\{-\frac{{\left[\left(P-\langle p\rangle \right)\cos\theta_{+}-\left(X-\langle x\rangle \right)\sin\theta_{+}\right]}^{2}}{(\sigma_{x}^{2}+\sigma_{p}^{2}-\Delta_{+})/N}\right\}, \end{array}$$

in which

(21) $$\begin{array}{ccc} \theta_{+} & = & \frac{1}{2}\arctan\left(\frac{2{\langle xp\rangle }_{c}}{\sigma_{x}^{2}-\sigma_{p}^{2}}\right), \end{array}$$

(22) $$\begin{array}{ccc} \Delta_{+} & = & \sqrt{{\left(\sigma_{x}^{2}-\sigma_{p}^{2}\right)}^{2}+4{\langle xp\rangle }_{c}^{2}}, \end{array}$$

(23) $$\begin{array}{ccc} {\langle xp\rangle }_{c} & = & \frac{1}{2}\langle \hat{x}\hat{p}+\hat{p}\hat{x}\rangle -\langle \hat{x}\rangle \langle \hat{p}\rangle . \end{array}$$

And the probability distribution for the imaginary parts $ℑ(c_{mn})$ is

(24) $$\begin{array}{ccc} P_{im}\left(X,P\right) & = & N_{1}\exp\left\{-\frac{{\left[\left(X-\langle x\rangle \right)\cos\theta_{-}+\left(P-\langle p\rangle \right)\sin\theta_{-}\right]}^{2}}{(\sigma_{x}^{2}+\sigma_{p}^{2}+\Delta_{-})/N}\right\}\\ & & \;\;\;\;\;\times \exp\left\{-\frac{{\left[\left(P-\langle p\rangle \right)\cos\theta_{-}-\left(X-\langle x\rangle \right)\sin\theta_{-}\right]}^{2}}{(\sigma_{x}^{2}+\sigma_{p}^{2}-\Delta_{-})/N}\right\}\\ & & -N_{2}\exp\left\{-\frac{{\left(P-\langle p\rangle \right)}^{2}}{2\sigma_{p}^{2}/N}-\frac{{\left(X-\langle x\rangle \right)}^{2}}{2\sigma_{x}^{2}/N}\right\}. \end{array}$$

In the above $N_{1}$ and $N_{2}$ are normalising factors, and

(25) $$\begin{array}{ccc} \theta_{-} & = & \frac{1}{2}\arctan\left(\frac{2{\langle xp\rangle }_{-}}{\sigma_{x}^{2}-\sigma_{p}^{2}}\right), \end{array}$$

(26) $$\begin{array}{ccc} \Delta_{-} & = & \sqrt{{\left(\sigma_{x}^{2}-\sigma_{p}^{2}\right)}^{2}+4{\langle xp\rangle }_{-}^{2}}, \end{array}$$

(27) $$\begin{array}{ccc} {\langle xp\rangle }_{-} & = & i\langle \hat{x}\hat{p}-\hat{p}\hat{x}\rangle . \end{array}$$

It is noted that the probability distribution for the imaginary parts, $P_{im}\left(X,P\right)$, explicitly contains the commutator of $\hat{x}$ and $\hat{p}$ in the quantities $\theta_{-}$ and $\Delta_{-}$. In fact, if $\hat{x}$ and $\hat{p}$ were commutative, then $\theta_{-}=0$ and we would have

(28) $$\begin{array}{ccc} P_{im}\left(X,P\right) & = & 0. \end{array}$$

For the probability distribution for the real parts, $P_{re}\left(X,P\right)$, we have a product of Gaussian distributions mixing combinations of the two generally non-commuting variables $\hat{X}$ and $\hat{P}$. However, if ${\langle xp\rangle }_{c}=0$, then we would have a factorisation into two Gaussian distributions in $\hat{X}$ and $\hat{P}$ separately.

As a special case, upon the substitution

(29) $$\begin{array}{c} \hat{P}\to {⊗}_{i=1}^{N}{\hat{1}}_{i} \end{array}$$

in (19), the probability distribution $P_{im}\left(X,P\right)$ vanishes and the remaining distribution $P_{re}\left(X,P\right)$ reduces to a product of distributions of a single variable in (8). Alternatively, we could obtain these same results as with (8) by letting n=0 in (19).

## 5. Emergence of Classicality

From the results of the last section, we can readily derive the following expectation values

(30) $$\begin{array}{ccc} \langle \Phi \left|\hat{X}\right|\Phi \rangle & = & \langle x\rangle ; \end{array}$$

and

(31) $$\begin{array}{ccc} \Sigma_{X}^{2} & \equiv & \langle \Phi \left|{\hat{X}}^{2}\right|\Phi \rangle -{\langle \Phi \left|\hat{X}\right|\Phi \rangle }^{2},\\ & \overset{N\to \infty }{∼} & \frac{1}{2N}\left(\sigma_{x}^{2}+\sigma_{p}^{2}+\Delta_{+}\cos\left(2\theta_{+}\right)\right),\\ & \overset{N\to \infty }{∼} & \sigma_{x}^{2}/N. \end{array}$$

Similarly,

(32) $$\begin{array}{ccc} \langle \Phi \left|\hat{P}\right|\Phi \rangle & = & \langle p\rangle ; \end{array}$$

and

(33) $$\begin{array}{ccc} \Sigma_{P}^{2} & \equiv & \langle \Phi \left|{\hat{P}}^{2}\right|\Phi \rangle -{\langle \Phi \left|\hat{P}\right|\Phi \rangle }^{2},\\ & \overset{N\to \infty }{∼} & \frac{1}{2N}\left(\sigma_{x}^{2}+\sigma_{p}^{2}-\Delta_{+}\cos\left(2\theta_{+}\right)\right),\\ & \overset{N\to \infty }{∼} & \sigma_{p}^{2}/N. \end{array}$$

Furthermore, it can be shown that the correlation between the coarse-grained/ renormalisation block variables $\hat{X}$ and $\hat{P}$

(34) $$\begin{array}{ccc} \frac{1}{2}\langle \Phi \left|\hat{X}\hat{P}+\hat{P}\hat{X}\right|\Phi \rangle & \overset{N\to \infty }{∼} & \int dXdP\;XP\;P_{re}\left(X,P\right),\\ & \overset{N\to \infty }{∼} & \langle x\rangle \langle p\rangle +{\langle xp\rangle }_{c}/N,\\ & \overset{N\to \infty }{∼} & \langle \Phi \left|\hat{X}\right|\Phi \rangle \langle \Phi \left|\hat{P}\right|\Phi \rangle +O\left(1/N\right), \end{array}$$

indicating that, in this limit, the coarse-grained/renormalisation block variables are uncorrelated and behaving as classically independent variables.

For the expectation value of the *Hermitian* commutator, $i\langle \Phi \left|\hat{X}\hat{P}-\hat{P}\hat{X}\right|\Phi \rangle$, we integrate (19) with the distribution $P_{im}\left(X,P\right)$ for the imaginary part (24) to obtain

(35) $$\begin{array}{ccc} i\langle \Phi \left|\hat{X}\hat{P}-\hat{P}\hat{X}\right|\Phi \rangle & \overset{N\to \infty }{∼} & \Delta_{-}\sin\left(2\theta_{-}\right)/2N={\langle xp\rangle }_{-}/N. \end{array}$$

It thus also follows that if $\langle \hat{x}\hat{p}-\hat{p}\hat{x}\rangle =0$, then $\langle \Phi \left|\hat{X}\hat{P}-\hat{P}\hat{X}\right|\Phi \rangle =0$, identically for any value of *N*.

We further observe that, in the limit of infinitely many identical and non-interacting quantum subsystems, N→∞,

(36) $$\begin{array}{ccc} P_{re}\left(X,P\right) & \overset{N\to \infty }{⟶} & \delta \left(\left(X-\langle x\rangle \right)\cos\theta_{+}+\left(P-\langle p\rangle \right)\sin\theta_{+}\right)\delta \left(\left(P-\langle p\rangle \right)\cos\theta_{+}-\left(X-\langle x\rangle \right)\sin\theta_{+}\right),\\ & \overset{N\to \infty }{⟶} & \delta \left(\left(X-\langle x\rangle \right)/\cos\theta_{+}\right)\delta \left(\left(P-\langle p\rangle \right)\cos\theta_{+}\right),\\ & \overset{N\to \infty }{⟶} & \delta (X-\langle x\rangle )\delta (P-\langle p\rangle ). \end{array}$$

and

(37) $$\begin{array}{ccc} P_{im}\left(X,P\right) & \overset{N\to \infty }{⟶} & \delta \left(\left(X-\langle x\rangle \right)\cos\theta_{-}+\left(P-\langle p\rangle \right)\sin\theta_{-}\right)\delta \left(\left(P-\langle p\rangle \right)\cos\theta_{-}-\left(X-\langle x\rangle \right)\sin\theta_{-}\right)\\ & & -\delta (X-\langle x\rangle )\delta (P-\langle p\rangle ),\\ & \overset{N\to \infty }{⟶} & 0. \end{array}$$

Thus,

(38) $$\begin{array}{ccc} \langle \Phi \left|\sum_{mn}\left(c_{mn}X^{m}P^{n}+c_{mn}^{*}P^{n}X^{m}\right)\right|\Phi \rangle & \overset{N\to \infty }{⟶} & 2\sum_{mn}ℜ\left(c_{mn}\right){\langle x\rangle }^{m}{\langle p\rangle }^{n}. \end{array}$$

The right-hand side above now only involves ${\langle x\rangle }^{i}$ and ${\langle p\rangle }^{j}$ (with some integers *i* and *j*), and contains neither $\langle x^{i}\rangle$ nor $\langle p^{j}\rangle$, nor the quantum correlations $\langle x^{i}p^{j}\rangle$. It is also implied in this last expression, which does not include the imaginary parts $ℑ(c_{mn})$, that the expectation value of the commutator of the coarse-grained/renormalisation block variables $\hat{X}$ and $\hat{P}$ is vanishingly small with a sufficiently large *N*, in agreement with (35).

In general, any *classical* observable can indeed be expressed as a restricted form of the left-hand side of (38) with a *real* $c_{mn}$—thus removing the need to consider the distribution for the imaginary part $P_{im}\left(X,P\right)$.

As a consequence, a regime of classicality emerges due to the fact that quantum correlations and all traces of quantum behaviours are now suppressed, except those inherent in the quantum expectation values 〈x〉 and 〈p〉.

## 6. Differential Entropies

A direct generalisation of information Shannon entropy for discrete probabilities $p_{d}$ [24]

(39) $$\begin{array}{ccc} S_{d} & = & -k_{B}\sum_{i}p_{d}^{\left(i\right)}\ln p_{d}^{\left(i\right)} \end{array}$$

to the case of continuous probability distributions might be

(40) $$\begin{array}{ccc} DEnt_{1} & = & -k_{B}\int Pr\left(X,P\right)\ln Pr\left(X,P\right)\;dXdP. \end{array}$$

Which is normally called the differential entropy.

This definition of differential entropy, however, does not share all properties of discrete entropy. For example, the differential entropy above can be negative; more importantly, however, it is not invariant under continuous coordinate transformations. In fact, Jaynes [22] showed that the expression above is not the correct limit of the expression for a finite set of probabilities.

He introduced a modification of differential entropy to address the defects in the initial definition of differential entropy by adding an invariant measure factor to correct this [22].

In information theory, this is the limiting density of discrete points in an adjustment to Shannon’s formula for differential entropy.

In the phase space volume $\Delta X_{i}\Delta P_{i}$, the transition from discrete probability to continuous probability density should be

(41) $$\begin{array}{ccc} p_{d}^{\left(i\right)} & \to & Pr\left(X_{i},P_{i}\right)\Delta X_{i}\Delta P_{i}. \end{array}$$

If this passage to the limit is sufficiently well behaved, we would have

(42) $$\begin{array}{ccc} \lim_{N_{X}\to \infty }\frac{1}{N_{X}}\left(\mathrm{number}\;\mathrm{of}\;\mathrm{points}\;\mathrm{in}\;\left[X_{i},X_{i}+\Delta X_{i}\right]\right) & = & \int_{X_{i}}^{X_{i}+\Delta X_{i}}m\left(X\right)dX, \end{array}$$

where $N_{X}$ is the number of points in the *X* dimension, and $m(X_{i})$ is the density in this dimension. As a result, the differences $\Delta X_{i}$ in the neighbourhood of any particular value of $X_{i}$ will have to be

(43) $$\begin{array}{c} \lim_{N_{X}\to \infty }N_{X}\Delta X_{i}={\left[m\left(X_{i}\right)\right]}^{-1}. \end{array}$$

We have, on the other hand, for the probability density

(44) $$\begin{array}{ccc} m(X_{i}) & = & \int Pr(X_{i},P)dP. \end{array}$$

Thus,

(45) $$\begin{array}{c} \lim_{N_{X}\to \infty }N_{X}\Delta X_{i}={\left[\int Pr(X_{i},P)dP\right]}^{-1}. \end{array}$$

Similarly,

(46) $$\begin{array}{c} \lim_{N_{P}\to \infty }N_{P}\Delta P_{i}={\left[\int Pr(X,P_{i})dX\right]}^{-1}. \end{array}$$

Combining all the above, we have

(47) $$\begin{array}{ccc} DiffEnt & = & \lim_{N_{X},\;N_{P}\to \infty }\\ & & -k_{B}\sum_{i}Pr\left(X_{i},P_{i}\right)\ln\left(\frac{Pr(X_{i},P_{i})}{N_{X}N_{P}\int Pr\left(X_{i},P\right)dP\int Pr\left(X,P_{i}\right)dX}\right)\Delta X_{i}\Delta P_{i},\\ & = & -k_{B}\int Pr\left(X,P\right)\ln\left(\frac{Pr(X,P)}{\int Pr(X,P)dP\int Pr(X,P)dX}\right)dXdP\\ & & +\lim_{N_{X},\;N_{P}\to \infty }k_{B}\ln\left(N_{X}N_{P}\right). \end{array}$$

Hereinafter, we adopt, following Jaynes, the above as a modified differential entropy, but without the second term, which is infinitely large in the limit, and without the minus sign for the first term to keep our entropy definition semi-positive,

(48) $$\begin{array}{ccc} DEnt & = & k_{B}\int Pr\left(X,P\right)\ln\left(\frac{Pr(X,P)}{\int Pr(X,P)dP\int Pr(X,P)dX}\right)dXdP. \end{array}$$

This entropy notion is a special instance of the relative entropy in information theory, also known as the Kullback–Leibler divergence [25] or relative entropy. It is a statistical distance to measure how one probability distribution is different from a second reference probability distribution. A simple interpretation of this divergence is the expected excess surprise from using the latter as a model when the actual distribution is the reference distribution.

We will now investigate the time dependence of such entropy for some systems of non-interacting components. It is noted, and will be illustrated in the next section, that it is the quantum origin of the non-factorisation of Pr(X,P) (19) into component distributions of *X* and *P* that gives rise to some of the interesting and non-trivial temporal behaviours of entropies.

## 7. Time-Dependent Entropies of Some Simple Systems

There are, in the literature, some considerations of so-called joint entropy for some simple quantum mechanical systems of a single particle [26,27]. In this paper, we subsequently consider, in contrast, certain entropies of composite systems when the number of constituents is infinitely large.

Restricting ourselves to observables in general, it suffices to consider only the particular case whereby $c_{mn}$ in (19) are real. Substituting the probability distribution for the real component (20) (which suffices for classical observables) into our adopted entropy (48), we arrive at

(49) $$\begin{array}{ccc} DEnt & = & -k_{B}\ln\left[\left(\sigma_{x}^{2}\sigma_{p}^{2}-{\langle xp\rangle }_{c}\right)/\sigma_{x}^{2}\sigma_{p}^{2}\right]. \end{array}$$

We see from this explicit expression that the non vanishing of ${\langle xp\rangle }_{c}$ in general, due to quantum correlations, enables some non-trivial time dependence for the differential entropy.

### 7.1. Free Particles

For free particles in one dimension, we have for the individual constituent, in the Heisenberg picture,

(50) $$\begin{array}{ccc} \hat{H} & = & \frac{{\hat{p}}^{2}}{2m},\\ \hat{x}\left(t\right) & = & \hat{x}\left(0\right)+\frac{\hat{p}\left(0\right)}{m}t,\\ \hat{p}\left(t\right) & = & \hat{p}\left(0\right)=\hat{p}. \end{array}$$

The time-dependent variance of the centre of mass, with finite initial variances $\sigma_{x}^{2}\left(0\right)$ and $\sigma_{p}^{2}\left(0\right)$, which assumes the following temporal behaviours:

(51) $$\begin{array}{ccc} \sigma_{p}\left(t\right) & = & \mathrm{constant}, \end{array}$$

and

(52) $$\begin{array}{ccc} \sigma_{x}^{2}\left(t\right) & = & \left(\sigma_{x}^{2}\left(0\right)+\frac{t^{2}}{m^{2}}\sigma_{p}^{2}+\frac{t}{m}\left(\langle \hat{x}\left(0\right)\hat{p}+\hat{p}\hat{x}\left(0\right)\rangle -2\langle \hat{x}\left(0\right)\rangle \langle \hat{p}\rangle \right)\right). \end{array}$$

It then follows that the coarse-grained entropy (49), for a sizable collection of *N*-free and -independent particles and for sufficiently large time, behaves as (*cf* Sackur–Tetrode equation for ideal gas)

(53) $$\begin{array}{c} DEnt\left(t\right)\overset{t\to \infty }{⟶}O\left(\ln|t|\right). \end{array}$$

Which is irreversibly increasing with time (unless the individual subsystem is initially in a momentum eigenstate, whereby $\sigma_{p}^{2}\left(0\right)=0={\langle x\left(0\right)p\left(0\right)\rangle }_{c}$). Our entropy is increasing with time although invariant with time-reversal, t→−t and $\hat{p}\to -\hat{p}$—as is the symmetry of the underlying dynamics of an individual constituent particle.

### 7.2. Uniform and Constant Force

For a system under a uniform and constant external force, we have in the Heisenberg picture

(54) $$\begin{array}{ccc} \hat{H} & = & \frac{{\hat{p}}^{2}}{2m}-a\hat{x},\\ \hat{x}\left(t\right) & = & \hat{x}\left(0\right)+\hat{p}\left(0\right)t/m+at^{2}/2m,\\ \hat{p}\left(t\right) & = & \hat{p}\left(0\right)+at. \end{array}$$

From which follows the time dependence

(55) $$\begin{array}{ccc} \sigma_{x}^{2}\left(t\right) & = & \left(\sigma_{x}^{2}\left(0\right)+\frac{t^{2}}{m^{2}}\sigma_{p}{\left(0\right)}^{2}+\frac{t}{m}\left(\langle \hat{x}\left(0\right)\hat{p}\left(0\right)+\hat{p}\left(0\right)\hat{x}\left(0\right)\rangle -2\langle \hat{x}\left(0\right)\rangle \langle \hat{p}\left(0\right)\rangle \right)\right), \end{array}$$

and

(56) $$\begin{array}{ccc} \sigma_{p}^{2}\left(t\right) & = & \sigma_{p}^{2}\left(0\right). \end{array}$$

Upon which, the coarse-grained entropy is, for a large amount of time, also irreversibly increasing,

(57) $$\begin{array}{ccc} DEnt & \overset{t\to \infty }{⟶} & O(\ln|t|), \end{array}$$

unless $\sigma_{p}^{2}\left(0\right)=0$ and ${\langle x\left(0\right)p\left(0\right)\rangle }_{c}=0$, that is, when the individual subsystem is in a momentum eigenstate initially. The initial position eigenstate is also not applicable here because that would imply an unbounded variance of the momentum due to a quantum uncertainty relation.

### 7.3. Oscillatory Particles

On the other hand, an example in which the differential entropy is not monotonic in time is that of the quantum simple harmonic oscillator,

(58) $$\begin{array}{ccc} \hat{H} & = & \frac{{\hat{p}}^{2}}{2m}+\frac{1}{2}m\omega^{2}{\hat{x}}^{2},\\ \hat{x}\left(t\right) & = & \hat{x}\left(0\right)\cos\left(\omega t\right)+\frac{\hat{p}\left(0\right)}{m\omega }\sin\left(\omega t\right),\\ \hat{p}\left(t\right) & = & \hat{p}\left(0\right)\cos\left(\omega t\right)-m\omega \hat{x}\left(0\right)\sin\left(\omega t\right). \end{array}$$

From which,

(59) $$\begin{array}{ccc} \sigma_{x}^{2}\left(t\right) & = & \cos^{2}\left(\omega t\right)\sigma_{x}^{2}\left(0\right)+\frac{\sin^{2}\left(\omega t\right)}{m^{2}\omega^{2}}\sigma_{p}^{2}\left(0\right)+2\frac{\cos(\omega t)\sin(\omega t)}{m\omega }{\langle \hat{x}\left(0\right)\hat{p}\left(0\right)\rangle }_{c}, \end{array}$$

and

(60) $$\begin{array}{ccc} \sigma_{p}^{2}\left(t\right) & = & \cos^{2}\left(\omega t\right)\sigma_{p}^{2}\left(0\right)+m^{2}\omega^{2}\sin^{2}\left(\omega t\right)\sigma_{x}^{2}\left(0\right)-2m\omega \cos(\omega t)\sin(\omega t){\langle \hat{x}\left(0\right)\hat{p}\left(0\right)\rangle }_{c}. \end{array}$$

In this case, the differential entropy (49) is not, even for a large time, an approximately monotonic function of time.

## 8. Summary and Concluding Remarks

We derive some quantum mechanical versions of central limit theorems for the expectation values of coarse-grained observables, which are functions of coarse-grained Hermitian operators. The coarse-grained variables considered above correspond to the centre of mass and its classical velocity.

Our derivation methodology could also be rephrased explicitly as a restricted form of renormalisation blocking applied only for observables, and not for non-Hermitian operators. Although incomplete in that sense, the restricted renormalisation is important and sufficiently useful for the consideration of all the bulk behaviours that are observable and measurable.

From such Hermiticity constraints, we obtain, for the expectation values of observables, positive-definite distributions, which are also the fixed points of the restricted renormalisation group flows. Our probability distributions are *unique* and *independent* of the operators being considered. Those are the results in (8) for the functions of single macroscopically coarse-grained variables and that in (19) for functions of macroscopically coarse-grained non-commutative quantum variables. In the latter, we have two separate distributions for the real and imaginary parts (20) and (24), respectively—even though we only need to consider the real part for observables.

Furthermore, our results herein could also be applied to systems of interacting constituents with approximation whereby the many-body problem could essentially be reduced to a one-body problem, like the mean field Hartree method.

Our probability distributions enable a pathway for the emergence of classical coarse-graining behaviours, as far as observable and measurable, in the limit of an infinitely large number of identical and non-interacting quantum constituents (having finite variances for relevant variables). This is the result of the fact that quantum correlations and all traces of quantum behaviours are now suppressed or cancelled out, as shown in (38), except those inherent in the 〈x〉 and 〈p〉 of the constituents.

It should be emphasised that this particular mechanism for such emergence is entirely due to coarse graining in the macroscopic limit, and not because of environmental decoherence, nor some kinds of interactions among the constituents, nor due an introduction of noises into the Schrödinger equation as with the GRWP approach. It is beyond the scope of this paper to review all the emergence mechanisms, particularly when there is no exclusivity of our CLT-induced mechanism.

It is important to note that, because the wave functions are generally time-dependent, in the derivation of the above results, we have had to work with a *same* time instant for all the microscopic constituent wave functions, as demonstrated in (A3). That is, expectation values of the different components ${\hat{x}}_{i}$ and ${\hat{p}}_{i}$ in the block variables $\hat{X}$ and $\hat{P}$ must be evaluated at the *same* time. This situation is in stark contrast to the classical central limit theorems, which, when dealing with time-independent iid components, can be employed for averaging measurement results over *different* moments in time. This distinction is important in our context to recognise that an emergence of classicality would only be applicable for macroscopically block variables—and not for microscopic variables repeatedly measured and averaged over time. The double-slit experiments could illustrate our point here. A single electron going one by one through the apparatus still exhibits interference, but macroscopic particles (a macroscopic bunch of many electrons at the same moment of time) may not.

The probability distributions of the quantum central limit theorem further allow us to evaluate some differential entropies for the composites of macroscopically coarse-grained systems. Those entropies are symmetric with respect to the time reversal (t→−t, p→−p and $\phi \to \phi^{*}$), as is the underlying quantum dynamics. Nevertheless, they could have some interesting and non-trivial temporal dependence. It is noted that it is the quantum origin of the non-factorisation of Pr(X,P) (19) into the product of component distributions of *X* and *P* that gives rise to some interesting and non-trivial temporal behaviours of the entropies. In fact, in some instances, they could increase with time approximately monotonically—as functions of the absolute value of the time and for a sufficiently large time. This is in stark contrast to the von Neumann entropy which simply vanishes on pure states.

As is the case of classical central limit theorems which have been generalised to cover some less stringent constraints on the behaviours of the constituent components [2], we expect that further quantum central limit theorems may also be similarly generalised.

## Appendix A. Derivation of a Quantum Central Limit Theorem for Function of a Coarse-Grained/Renormalisation Block Variable

We derive the result (8) in this Appendix.

Consider a system with identical and non-interacting components (as in the case of an ideal gas)

$$|\Phi \rangle ={⊗}_{i}^{N}|\phi_{i}\rangle ,$$

where $|\phi_{i}\rangle =|\phi \rangle$, for all *i*.

Now with some function *f*, we consider

(A1) $$\begin{array}{ccc} E & = & \langle \Phi \left|f\left(\frac{1}{N}\sum_{i}^{N}{\hat{x}}_{i}\right)\right|\Phi \rangle \end{array}$$

Insert the resolution of identity into the above

$$\hat{1}=\int \prod_{i}^{N}dx_{i}|x_{i}\rangle \langle x_{i}|,$$

where

$$\begin{array}{ccc} {\hat{x}}_{i}|y_{i}\rangle & = & y_{i}|y_{i}\rangle . \end{array}$$

Then we have

(A2) $$\begin{array}{ccc} E & = & \langle \Phi \left|\left(\int \prod_{j}^{N}dy_{j}|y_{j}\rangle \langle y_{i}|\right)\int f\left(\frac{1}{N}\sum_{i}^{N}{\hat{x}}_{i}\right)\prod_{k}^{N}dx_{k}|x_{k}\rangle \langle x_{k}|\right|\Phi \rangle \\ & = & \int f\left(\frac{1}{N}\sum_{i}^{N}x_{i}\right)\prod_{k}^{N}{\left|\langle x_{k}|\phi_{k}\rangle \right|}^{2}dx_{k} \end{array}$$

Insert the identity

$$1=\int dX\;\delta \left(X-\frac{1}{N}\sum_{j}^{N}x_{j}\right),$$

in which the delta function can be expressed as

$$\delta \left(u\right)=\frac{1}{2\pi }\int dw\;e^{iwu}.$$

We further obtain

(A3) $$\begin{array}{ccc} E & = & \int dX\int \delta \left(X-\frac{1}{N}\sum_{j}^{N}x_{j}\right)f\left(\frac{1}{N}\sum_{k}^{N}x_{k}\right)\prod_{i}^{N}{\left|\langle x_{i}|\phi_{i}\rangle \right|}^{2}dx_{i},\\ & = & \frac{1}{2\pi }\int dX\;dw\;f\left(X\right)\;e^{iXw}{\left[\int dx\;e^{-ixw/N}{\left|\langle x|\phi \rangle \right|}^{2}\right]}^{N}, \end{array}$$

It should be emphasised that the probability distributions of the constituents $|\langle x|\phi_{i}\rangle {|}^{2}$ are functions of the time in general; and that, in arriving at (A3), we have had to take the *same* instant of time for all the component probabilities.

(A4) $$\begin{array}{ccc} E & = & \frac{1}{2\pi }\int dX\;dw\;f\left(X\right)\;e^{iXw}{\left[\int dx\;\left(1-iwx/N-w^{2}x^{2}/2N^{2}+O\left(1/N^{3}\right)\right){\left|\langle x|\phi \rangle \right|}^{2}\right]}^{N},\\ & = & \frac{1}{2\pi }\int dX\;dw\;f\left(X\right)\;e^{iXw}{\left[\;1-iw\langle x\rangle /N-w^{2}\langle x^{2}\rangle /2N^{2}+O\left(1/N^{3}\right)\right]}^{N},\\ & = & \frac{1}{2\pi }\int dX\;dw\;f\left(X\right)\;e^{iXw}\;\exp\left\{-iw\langle x\rangle -w^{2}\left(\langle x^{2}\rangle -{\langle x\rangle }^{2}\right)/2N+O\left(1/N^{2}\right)\right\}. \end{array}$$

Integrating over *w*, we finally arrive at the result

(A5) $$\begin{array}{ccc} \langle \Phi \left|f\left(\frac{1}{N}\sum_{i}^{N}{\hat{x}}_{i}\right)\right|\Phi \rangle & \overset{N\to \infty }{⟶} & \frac{1}{\left(\sigma_{x}/\sqrt{N}\right)2\pi \sqrt{2\pi }}\int dXf\left(X\right)\exp\left\{-\frac{{\left(X-\langle x\rangle \right)}^{2}}{2{\left(\sigma_{x}/\sqrt{N}\right)}^{2}}\right\}, \end{array}$$

where in (A4) and (A5), we have defined

(A6) $$\begin{array}{ccc} \langle x\rangle & \equiv & {\int x|\langle x|\phi \rangle |}^{2}dx=\langle \phi |\hat{x}|\phi \rangle , \end{array}$$

(A7) $$\begin{array}{ccc} \sigma_{x}^{2} & \equiv & \langle x^{2}\rangle -{\langle x\rangle }^{2}. \end{array}$$

## Appendix B. Derivation of a Quantum Central Limit Theorem for Non-Commuting Operators

In this appendix, we derive the result (19) for our quantum central limit theorem.

Let us first consider the expectation value,

(A8) $$\begin{array}{ccc} G & = & \langle \Phi \left|{\left(\hat{X}\right)}^{m}{\left(\hat{P}\right)}^{n}+{\left(\hat{P}\right)}^{n}{\left(\hat{X}\right)}^{m}\right|\Phi \rangle ,\\ & = & \langle \Phi \left|\left(\int \prod_{i}^{N}dy_{i}|y_{i}\rangle \langle y_{i}|\right){\left(\hat{X}\right)}^{m}{\left(\hat{P}\right)}^{n}\left(\int \prod_{i}^{N}dk_{i}|k_{i}\rangle \langle k_{i}|\right)\right.+\\ & & \left.+\left(\int \prod_{i}^{N}dk_{i}|k_{i}\rangle \langle k_{i}|\right){\left(\hat{P}\right)}^{n}{\left(\hat{X}\right)}^{m}\left(\int \prod_{i}^{N}dy_{i}|y_{i}\rangle \langle y_{i}|\right)\right|\Phi \rangle ,\\ & = & \int {\left(\sum_{i}^{N}y_{i}/N\right)}^{m}{\left(\sum_{i}^{N}k_{i}/N\right)}^{n}\\ & & \left(\prod_{i}^{N}\langle \phi_{i}|y_{i}\rangle \langle y_{i}|k_{i}\rangle \langle k_{i}|\phi_{i}\rangle dy_{i}dk_{i}+\prod_{i}^{N}\langle \phi_{i}|k_{i}\rangle \langle k_{i}|y_{i}\rangle \langle y_{i}|\phi_{i}\rangle dy_{i}dk_{i}\right), \end{array}$$

where we insert the resolutions of identity, respectively, for the *x* and *p* representation,

(A9) $$\begin{array}{c} \hat{1}=\int \prod_{i}^{N}dy_{i}|y_{i}\rangle \langle y_{i}|, \end{array}$$

(A10) $$\begin{array}{c} \hat{1}=\int \prod_{i}^{N}dk_{i}|k_{i}\rangle \langle k_{i}|, \end{array}$$

in which the eigenvectors of position and momentum, respectively, satisfy

(A11) $$\begin{array}{ccc} {\hat{x}}_{i}|y_{i}\rangle & = & y_{i}|y_{i}\rangle , \end{array}$$

(A12) $$\begin{array}{ccc} {\hat{p}}_{i}|k_{i}\rangle & = & k_{i}|k_{i}\rangle . \end{array}$$

We then insert to integrand of (A8), and the identities

(A13) $$\begin{array}{c} 1=\int dX\;\delta \left(X-\frac{1}{N}\sum_{j}^{N}y_{j}\right)=\int dP\;\delta \left(P-\frac{1}{N}\sum_{j}^{N}k_{j}\right), \end{array}$$

in which the delta functions can also be expressed as

(A14) $$\begin{array}{c} \delta \left(X-\sum_{j}^{N}y_{j}/N\right)=\frac{1}{2\pi }\int dw\;e^{iw(X-\sum_{j}^{N}y_{j}/N)}, \end{array}$$

(A15) $$\begin{array}{c} \delta \left(P-\sum_{j}^{N}p_{j}/N\right)=\frac{1}{2\pi }\int d\lambda \;e^{i\lambda (P-\sum_{j}^{N}p_{j}/N)}. \end{array}$$

From (A8), we then have

(A16) $$\begin{array}{ccc} G & = & \frac{1}{2}\int dXdP\;\delta \left(X-\frac{1}{N}\sum_{j}^{N}y_{j}\right)\delta \left(P-\frac{1}{N}\sum_{j}^{N}k_{j}\right)g\left(\frac{1}{N}\sum_{i}^{N}y_{i},\frac{1}{N}\sum_{i}^{N}k_{i}\right)\\ & & \left(\prod_{i}^{N}\langle \phi_{i}|y_{i}\rangle \langle y_{i}|k_{i}\rangle \langle k_{i}|\phi_{i}\rangle dy_{i}dk_{i}+\prod_{i}^{N}\langle \phi_{i}|k_{i}\rangle \langle k_{i}|y_{i}\rangle \langle y_{i}|\phi_{i}\rangle dy_{i}dk_{i}\right),\\ & = & \frac{1}{8\pi^{2}}\int dXdP\;dwd\lambda \;g\left(X,P\right)\;e^{iXw+iP\lambda }\\ & & \left[{\left(\int dydk\;e^{-iyw/N-ik\lambda /N}\langle \phi |y\rangle \langle y|k\rangle \langle k|\phi \rangle \right)}^{N}+{\left(\int dydk\;e^{-iyw/N-ik\lambda /N}\langle \phi |k\rangle \langle k|y\rangle \langle y|\phi \rangle \right)}^{N}\right],\\ & = & \frac{1}{8\pi^{2}}\int dXdP\;dwd\lambda \;g\left(X,P\right)\;e^{iXw+iP\lambda }\\ & & \left[{\left(\int dydk\;\left(1-i\left(wy+\lambda k\right)/N-{\left(wy+\lambda k\right)}^{2}/2N^{2}+O\left(1/N^{3}\right)\right)\langle \phi |y\rangle \langle y|k\rangle \langle k|\phi \rangle \right)}^{N}\right.\\ & & +\left.{\left(\int dydk\;\left(1-i\left(wy+\lambda k\right)/N-{\left(wy+\lambda k\right)}^{2}/2N^{2}+O\left(1/N^{3}\right)\right)\langle \phi |k\rangle \langle k|y\rangle \langle y|\phi \rangle \right)}^{N}\right],\\ & = & \frac{1}{8\pi^{2}}\int dXdP\;dwd\lambda \;g\left(X,P\right)\;e^{iXw+iP\lambda }\\ & & \left[\;{\left(1-iw\bar{y}/N-i\lambda \bar{k}/N-\bar{{\left(wy+\lambda k\right)}^{2}}/2N^{2}+O\left(1/N^{3}\right)\right)}^{N}+\right.\\ & & \left.+{\left(1-iw\tilde{y}/N-i\lambda \tilde{k}/N-\tilde{{\left(wy+\lambda k\right)}^{2}}/2N^{2}+O\left(1/N^{3}\right)\right)}^{N}\right],\\ & = & \frac{1}{8\pi^{2}}\int dXdP\;dwd\lambda \;g\left(X,P\right)\;e^{iXw+iP\lambda }\\ & & \left[\exp\left\{-iw\bar{y}-i\lambda \bar{k}-\left(\bar{{\left(wy+\lambda k\right)}^{2}}-{\left(w\bar{y}+\lambda \bar{k}\right)}^{2}\right)/2N+O\left(1/N^{2}\right)\right\}+\right.,\\ & & \left.+\exp\left\{-iw\tilde{y}-i\lambda \tilde{k}-\left(\tilde{{\left(wy+\lambda k\right)}^{2}}-{\left(w\tilde{y}+\lambda \tilde{k}\right)}^{2}\right)/2N+O\left(1/N^{2}\right)\right\}\right]. \end{array}$$

In the last two expressions, we introduce the notations

(A17) $$\begin{array}{ccc} \bar{c(y,k)} & \equiv & \int dydk\;c(y,k)\;\langle \phi |y\rangle \langle y|k\rangle \langle k|\phi \rangle , \end{array}$$

(A18) $$\begin{array}{ccc} \tilde{c(y,k)} & \equiv & \int dydk\;c(y,k)\;\langle \phi |k\rangle \langle k|y\rangle \langle y|\phi \rangle . \end{array}$$

It can be seen that, for any function a(y) of the position *y*, using the completeness of the momentum basis,

(A19) $$\begin{array}{ccc} \bar{a(y)} & = & \int dydk\;a(y)\;\langle \phi |y\rangle \langle y|k\rangle \langle k|\phi \rangle ,\\ & = & \int dy\;a\left(y\right)\;\langle \phi |y\rangle \langle y|\left(\int dk|k\rangle \langle k|\right)|\phi \rangle ,\\ & = & \int dy\;a(y)\;\langle \phi |y\rangle \langle y|\phi \rangle ,\\ \bar{a(y)} & = & \langle a\left(\hat{y}\right)\rangle . \end{array}$$

Similarly,

(A20) $$\begin{array}{ccc} \tilde{a(y)} & = & \int dydk\;a(y)\;\langle \phi |k\rangle \langle k|y\rangle \langle y|\phi \rangle ,\\ & = & \int dy\;a\left(y\right)\;\langle \phi |\left(\int dk|k\rangle \langle k|\right)|y\rangle \langle y|\phi \rangle ,\\ & = & \int dy\;a(y)\;\langle \phi |y\rangle \langle y|\phi \rangle ,\\ \tilde{a(y)} & = & \langle a\left(\hat{y}\right)\rangle . \end{array}$$

Thus,

(A21) $$\begin{array}{ccc} \bar{a(y)} & = & \tilde{a(y)}=\langle a\left(\hat{y}\right)\rangle . \end{array}$$

Furthermore, for any function b(k),

(A22) $$\begin{array}{ccc} \bar{b(k)} & = & \tilde{b(k)}=\langle b\left(\hat{k}\right)\rangle . \end{array}$$

For a product of $a\left(\hat{y}\right)b\left(\hat{k}\right)$,

$$\begin{array}{ccc} \langle a\left(\hat{y}\right)b\left(\hat{k}\right)\rangle & = & \langle \phi |a\left(\hat{y}\right)b\left(\hat{k}\right)|\phi \rangle ,\\ & = & \langle \phi |\left(\int dy|y\rangle \langle y|\right)a\left(\hat{y}\right)b\left(\hat{k}\right)\left(\int dk|k\rangle \langle k|\right)|\phi \rangle ,\\ & = & \int dydk\;a(y)b(k)\;\langle \phi |y\rangle \langle y|k\rangle \langle k|\phi \rangle , \end{array}$$

that is,

(A23) $$\begin{array}{ccc} \bar{a(y)b(k)} & = & \langle a\left(\hat{y}\right)b\left(\hat{k}\right)\rangle . \end{array}$$

On the other hand, for the reverse ordering, it also follows that

(A24) $$\begin{array}{ccc} \tilde{a(y)b(k)} & = & \langle b\left(\hat{k}\right)a\left(\hat{y}\right)\rangle . \end{array}$$

We now introduce the notation

(A25) $$\begin{array}{ccc} {\langle xp\rangle }_{c} & = & \frac{1}{2}\langle \hat{x}\hat{p}+\hat{p}\hat{x}\rangle -\langle \hat{x}\rangle \langle \hat{p}\rangle . \end{array}$$

Back to (A16), we can rewrite that expression as

(A26) $$\begin{array}{ccc} G & ∼ & \frac{1}{2}\int dXdP\;dwd\lambda \;g\left(X,P\right)\;e^{iXw+iP\lambda -iw\langle y\rangle -i\lambda \langle k\rangle }\\ & & \left[\exp\left\{-\left(w^{2}\sigma_{y}^{2}+\lambda^{2}\sigma_{k}^{2}+2w\lambda \left(\langle \hat{y}\hat{k}\rangle -\langle \hat{y}\rangle \langle \hat{k}\rangle \right)\right)/2N+O\left(1/N^{2}\right)\right\}+\right.\\ & & \left.+\exp\left\{-\left(w^{2}\sigma_{y}^{2}+\lambda^{2}\sigma_{k}^{2}+2w\lambda \left(\langle \hat{k}\hat{y}\rangle -\langle \hat{y}\rangle \langle \hat{k}\rangle \right)\right)/2N+O\left(1/N^{2}\right)\right\}\right].\\ & ∼ & \int dXdP\;dwd\lambda \;g\left(X,P\right)\;e^{iXw+iP\lambda -iw\langle y\rangle -i\lambda \langle k\rangle }\\ & & \exp\left\{-\left(w^{2}\sigma_{y}^{2}+\lambda^{2}\sigma_{k}^{2}+2w\lambda {\langle yk\rangle }_{c}\right)/2N+O\left(1/N^{2}\right)\right\}. \end{array}$$

Integrating the last expression over *w* and λ, we finally obtain, for N≫1,

(A27) $$\begin{array}{ccc} \langle \Phi \left|{\left(\hat{X}\right)}^{m}{\left(\hat{P}\right)}^{n}+{\left(\hat{P}\right)}^{n}{\left(\hat{X}\right)}^{m}\right|\Phi \rangle \\ \overset{N\to \infty }{∼} & & 2\int dXdP\;X^{m}P^{n}\\ & & \times \exp\left\{-\frac{{\left[\left(X-\langle x\rangle \right)\cos\theta_{+}+\left(P-\langle p\rangle \right)\sin\theta_{+}\right]}^{2}}{(\sigma_{x}^{2}+\sigma_{p}^{2}+\Delta_{+})/N}\right\}\\ & & \times \exp\left\{-\frac{{\left[\left(P-\langle p\rangle \right)\cos\theta_{+}-\left(X-\langle x\rangle \right)\sin\theta_{+}\right]}^{2}}{(\sigma_{x}^{2}+\sigma_{p}^{2}-\Delta_{+})/N}\right\}, \end{array}$$

where

(A28) $$\begin{array}{ccc} \theta_{+} & = & \frac{1}{2}\arctan\left(\frac{2{\langle xp\rangle }_{c}}{\sigma_{x}^{2}-\sigma_{p}^{2}}\right), \end{array}$$

(A29) $$\begin{array}{ccc} \Delta_{+} & = & \sqrt{{\left(\sigma_{x}^{2}-\sigma_{p}^{2}\right)}^{2}+4{\langle xp\rangle }_{c}^{2}}, \end{array}$$

(A30) $$\begin{array}{ccc} {\langle xp\rangle }_{c} & = & \frac{1}{2}\langle \hat{x}\hat{p}+\hat{p}\hat{x}\rangle -\langle \hat{x}\rangle \langle \hat{p}\rangle . \end{array}$$

This is the probability distribution for the real part $P_{re}\left(X,P\right)$ of (20).

Similarly to the derivation above, it can also be shown that the distribution for the imaginary part $P_{im}\left(X,P\right)$ of (24) is, for some integers *m* and *n*,

(A31) $$\begin{array}{ccc} i\langle \Phi \left|{\hat{X}}^{m}{\hat{P}}^{n}-{\hat{P}}^{n}{\hat{X}}^{m}\right|\Phi \rangle & \overset{N\to \infty }{∼} & \int dXdP\;X^{m}P^{n}\\ & & \left(N_{1}\exp\left\{-\frac{{\left[\left(X-\langle x\rangle \right)\cos\theta_{-}+\left(P-\langle p\rangle \right)\sin\theta_{-}\right]}^{2}}{(\sigma_{x}^{2}+\sigma_{p}^{2}+\Delta_{-})/N}\right\}\right.\\ & & \;\;\;\;\;\times \exp\left\{-\frac{{\left[\left(P-\langle p\rangle \right)\cos\theta_{-}-\left(X-\langle x\rangle \right)\sin\theta_{-}\right]}^{2}}{(\sigma_{x}^{2}+\sigma_{p}^{2}-\Delta_{-})/N}\right\}\\ & & -\left.N_{2}\exp\left\{-\frac{{\left(P-\langle p\rangle \right)}^{2}}{2\sigma_{p}^{2}/N}-\frac{{\left(X-\langle x\rangle \right)}^{2}}{2\sigma_{x}^{2}/N}\right\}\right), \end{array}$$

where

(A32) $$\begin{array}{ccc} \theta_{-} & = & \frac{1}{2}\arctan\left(\frac{2{\langle xp\rangle }_{-}}{\sigma_{x}^{2}-\sigma_{p}^{2}}\right), \end{array}$$

(A33) $$\begin{array}{ccc} \Delta_{-} & = & \sqrt{{\left(\sigma_{x}^{2}-\sigma_{p}^{2}\right)}^{2}+4{\langle xp\rangle }_{-}^{2}}, \end{array}$$

(A34) $$\begin{array}{ccc} {\langle xp\rangle }_{-} & = & i\langle \hat{x}\hat{p}-\hat{p}\hat{x}\rangle . \end{array}$$

We could recover the results from (8) by either putting n=0 or replacing $\hat{P}$ by $\hat{1}$ in (19).
